# Effects of Acyclovir and IVIG on Behavioral Outcomes after HSV1 CNS Infection

**DOI:** 10.1155/2017/5238402

**Published:** 2017-11-19

**Authors:** Chandran Ramakrishna, Mari S. Golub, Abby Chiang, Teresa Hong, Markus Kalkum, Edouard M. Cantin

**Affiliations:** ^1^Department of Molecular Immunology, City of Hope Beckman Research Institute, Duarte, CA, USA; ^2^Department of Environmental Toxicology, UC Davis, Davis, CA, USA

## Abstract

Herpes simplex virus 1 (HSV) encephalitis (HSE) has serious neurological complications, involving behavioral and cognitive impairments that cause significant morbidity and a reduced quality of life. We showed that HSE results from dysregulated central nervous system (CNS) inflammatory responses. We hypothesized that CNS inflammation is casually involved in behavioral abnormalities after HSE and that treatment with ACV and pooled human immunoglobulin (IVIG), an immunomodulatory drug, would improve outcomes compared to mice treated with phosphate buffered saline (PBS) or ACV alone. Anxiety levels were high in HSV-infected PBS and ACV-treated mice compared to mice treated with ACV + IVIG, consistent with reports implicating inflammation in anxiety induced by lipopolysaccharide (LPS) or stress. Female, but not male, PBS-treated mice were cognitively impaired, and unexpectedly, ACV was protective, while the inclusion of IVIG surprisingly antagonized ACV's beneficial effects. Distinct serum proteomic profiles were observed for male and female mice, and the antagonistic effects of ACV and IVIG on behavior were paralleled by similar changes in the serum proteome of ACV- and ACV + IVIG-treated mice. We conclude that inflammation and other factors mediate HSV-induced behavioral impairments and that the effects of ACV and IVIG on behavior involve novel mechanisms.

## 1. Introduction

Herpes simplex virus type 1 is a highly contagious neurotropic alpha herpesvirus with global prevalence estimates for latent infections exceeding 85% for adults 60 and older [1]. Latent HSV resides in sensory ganglionic neurons and other synaptically contiguous CNS neurons in a nonreplicating, transcriptionally repressed state. HSV is the major cause of sporadic encephalitis resulting from primary infections in newborns and/or from reactivation of latent virus in adults. While vastly improved diagnostic procedures combined with safe and effective antiviral therapies have significantly improved outcomes, mortality (~25%) and the incidence of debilitating neurological disabilities in survivors remain unacceptably high [2–4]. Typically, HSE is focal, involving the medial temporal lobe, and it usually runs an acute course [5]. Anatomically and functionally, long-term damage from HSE is usually confined to the limbic system though the reason is unclear, but speculation is that it relates to HSV entering the CNS via the olfactory bulb and/or the trigeminal nerve or possibly to some specific permissiveness or affinity of the limbic cortices for HSV [6–8]. Following intranasal inoculation of mice, HSV spreads via the olfactory and trigeminal routes to the piriform cortex, thalamus, amygdaloid nucleus, and medial hypothalamic nuclei which comprise the limbic region of the brain that is known to support a variety of functions including memory, emotion, behavior, and olfaction [9–13]. Importantly, inflammatory lesions and prolonged inflammatory infiltrates in these brain areas have been correlated with memory impairment in untreated mice surviving HSV infection [13], which establishes the utility of this model of HSE for exploring immunomodulatory treatments that could ameliorate long-term neurological deficits in humans as prolonged antiviral treatment failed to improve outcomes [14].

We have shown unequivocally that the CNS pathology underlying HSE arises from exaggerated CNS inflammatory responses rather than direct virus cytopathology. Importantly, inhibiting HSV replication with ACV after CNS inflammatory responses were initiated could not prevent mortality, because this failed to restrain the escalating inflammatory response [15, 16]. In contrast, treatment with IVIG at 24 h post infection (pi) prevented HSE in all infected mice by a mechanism shown to depend on IVIG's potent anti-inflammatory and immunomodulatory activities, rather than on virus neutralization [17]. In addition to being used as replacement therapy for treating primary and secondary immune deficiencies, IVIG use has expanded to include treatment of various autoimmune and inflammatory diseases. IVIG is food and drug administration (FDA) licensed for treating immune thrombocytopenia (ITP), Kawasaki's disease, Guillain-Barre syndrome and chronic inflammatory demyelinating polyneuropathy. IVIG's benefit in these diseases derives from its potent anti-inflammatory and immunomodulatory activities, which also account for the dramatic increase in off-label uses to include multiple sclerosis (MS) and Alzheimer's disease (discussed in [18, 19]). IVIG appears to have multiple mechanism(s) of action that may be disease specific. A recent novel mechanism proposes that sialylated IgGs (sIgGs) present in limiting amounts in IVIG preferentially bind the human C-type lectin receptor DC-SIGN or SIGNR1, the mouse homologue. SIGNR1 binding elicits IL-33 production from macrophages/DCs resulting in IL-4 secretion by basophils that leads to upregulation of the inhibition of FcR*γ*IIb concomitant with downregulation of the activating FcRs on innate cells, which results in protective anti-inflammatory responses in some mouse autoimmune disease models [20–24].

Consistent with HSE being an inflammatory disease, we reported that IVIG prevented development of HSE by exerting potent IL-10-dependent immunomodulatory effects that included modulating production and infiltrating of Ly6C^high^ inflammatory monocytes into the CNS and induction of regulatory T cell populations [17]. Notably, we showed that IVIG protection was independent of HSV-neutralizing antibodies and sIgGs and further discussed that low-dose IVIG was protective [17]. Based on these results, we designed experiments to validate the hypothesis that treatment of HSV-infected mice with combination antiviral therapy incorporating IVIG, a potent, widely used, FDA-licensed anti-inflammatory biological, would result in significantly improved neurological function in survivors compared to mice treated with ACV alone, the standard-of-care treatment. Our results show surprisingly that IVIG exerted both beneficial and deleterious effects, whereas ACV was either beneficial or neutral depending on the nature of the behavioral deficit. These unexpected results suggest that the effects of ACV and IVIG are complex and may include modulation of physiological pathways that impact behavior.

## 2. Materials and Methods

### 2.1. Mice, Virus, and Virus Inoculation

All animal procedures were performed in compliance with the City of Hope Institutional Animal Care and Use Committee (IACUC) and within the framework of the Guide for the Care and Use of Laboratory Animals. Mice on the C57BL6/J background (B6.129(cg)-Gt(ROSA)26Sor^tm4(ACTB-tdTomato-EGFP)Luo^/J mice) obtained from Jackson Laboratories, Bar Harbor, Maine, were bred in the vivarium at the City of Hope.

Master stocks of HSV1 strain 17^+^ comprised only of cell-released virus were prepared and titered using mycoplasma-free CV-1 cell monolayers as previously described [25]. Single-use aliquots of virus in Hanks balanced salt solution supplemented with 2% fetal bovine serum were stored at −80°C. Male and female mice, 6–8 weeks of age, were infected with HSV1 17^+^, a neurovirulent strain. Mice were sedated with ketamine (60 mg/kg) and xylazine (5 mg/kg) prior to HSV inoculation by corneal scarification. 129 mice were inoculated in one eye with 3200 PFU (equivalent to 10 × LD_50_) while B6 mice were bilaterally inoculated with 1 × 10^5^ plaque forming units (PFU) per eye and monitored daily as previously described [25]. Infectious virus in trigeminal ganglia (Tg), brain, and brainstem (BS) homogenates was determined by plaque assay [25]. Briefly, dilutions of cell-free homogenates are plated on Vero cells; the plates are incubated at 37°C with a growth medium supplemented with 2% human serum that contains HSV-neutralizing antibodies to prevent secondary plaque development. After incubation for 3-4 days at 37°C to allow plaques to grow, the monolayers are stained with crystal violet and the visualized plaques are counted under a microscope, which allows calculation of the virus titer as PFU/mL.

To increase the sensitivity of detection of infectious virus in brain tissues, monolayers that were negative in the initial screen were harvested and freeze-thawed 3 times to release intracellular infectious HSV and cell-free supernatants were then rescreened by repeat plaque assay.

### 2.2. Administration of Acyclovir and Intravenous Immunoglobulins

**Figure 1:**
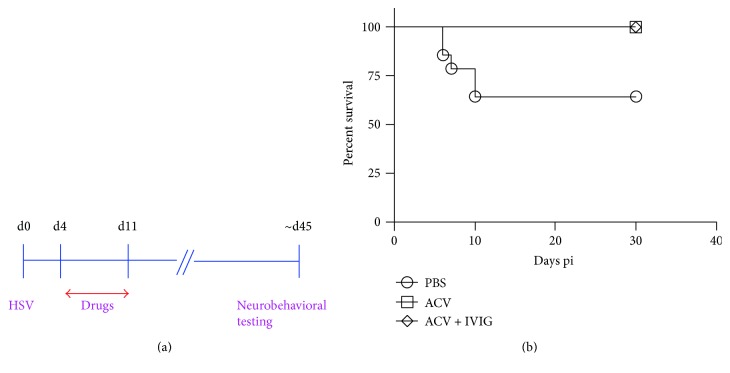
B6 mice bilaterally inoculated with 1 × 10^5^ PFU/eye then treated with PBS, ACV and ACV + IVIG according to the scheme in (a) were monitored for survival (b), *n* = 10–14 mice per group.

ACV obtained from APP Pharmaceuticals, Schuamburg, IL, was given at 50 mg/kg of body weight by intraperitoneal (ip) injection daily for 7 days starting on day 4 pi, and PBS was given according to the same schedule to control mice. IVIG (Carimune, NF) obtained from CSL Behring (King of Prussia, PA, USA) was given ip as a single 0.5 mL dose (25 mg/mouse) on day 4 pi in combination with a 7-day course of ACV according to the scheme in Figure 1(a).

### 2.3. Isolation of Mononuclear Cells from BS of HSV1-Infected Mice

CNS-derived mononuclear cells were isolated as previously described [17]. Briefly, brains and brainstem (BS) were removed separately from mice perfused with PBS, minced and digested with collagenase and DNAse for 30 min prior to centrifugation (800 g × 30 min) on a two-step Percol gradient. Brain refers to the whole brain minus the brainstem. The enriched population contained CNS infiltrating CD45^high^ leukocytes, CD45^int^ microglia, and CD45^neg^ CNS resident glial cells. CD45^high^ cells comprised approximately 5–8% of the total mononuclear cells isolated from the BS of naïve 129 wild-type (WT) mice. Cell viability was greater than 95% as revealed by trypan blue staining.

### 2.4. Flow Cytometry

Single-cell suspensions isolated from either, brain, BS, spleen, or cervical lymph nodes (CLN) were blocked with a 10% mixture of normal mouse, rat and horse serum, and rat anti-mouse CD16/32 (2.4G2, BD PharMingen) for 15 min prior to incubation with antibodies (Abs) to determine cell surface expression of various markers. Phycoerythrin (PE), FITC, and allophycocyanin-conjugated Abs specific for F480 (BM8), CD11b (M1/70), CD8 (53-6.7), and CD4 (RM4-4) were obtained from eBioscience (San Diego, CA). FITC, PE, and PerCP-conjugated Ly6-G (1A8), Ly6-C (AL-21), and CD45 (30-F11) were obtained from BD PharMingen (San Jose, CA). All isotype controls were obtained from eBioscience. To determine cell surface expression, antibody-labeled cells were acquired on a BD Fortessa Analyzer (BD Biosciences, San Jose, CA) and flow cytometry analysis was performed using FlowJo software (Tree Star Inc.). Doublets were excluded from live cell populations. CD45 was used to distinguish bone marrow-derived CD45^high^ leukocytes, from CD45^int^ CD11b^+^ microglia and CD45^neg^ neural/glial cells. Leukocyte subsets, calculated as a fraction of the live CD45^high^-infiltrating population in the BS, were expressed as percentages.

### 2.5. Detection of Cytokines and Chemokines

Serum samples collected on day 7 pi from two male and two female mice in each PBS, the ACV and ACV + IVIG groups were pooled (*n* = 4 mice/group) and analyzed for cytokines and chemokines using a ProCarta Mouse cytokine and chemokine 36plex luminex multibead array kit using a Bio-Rad Bio-Plex100 HTF System (eBioscience Inc., San Diego, CA) in the Clinical Immunobiology Correlative Studies Laboratory Core at the City of Hope, Duarte, CA.

### 2.6. Quantitative Proteomics Using the Tandem Mass Tag (TMT) Method

#### 2.6.1. Mouse Groups and Serum Samples

Sera from the following groups of C57BL/6 mice were used in the experiments: on day 0 (before infection), 2 females and 3 males; day 2 (post infection), 2 females and 3 males; day 7 (ACV treatment), 3 male and 2 female; day 7(ACV + IVIG treatment), 3 females and 2 males; and day 7 (PBS, mock treatment), 2 males and 3 females. Sera from each group were pooled separately for each sex, resulting in 5 pools from male and 5 pools from female mice.

#### 2.6.2. Serum Depletion

Albumin and immunoglobulins were separately removed from pooled sera of each gender subgroup using the Qproteome Murine Albumin Depletion Kit (Qiagen Inc., Valencia, CA) according to the manufacturer's protocol. Briefly, aliquots of 25 *μ*L serum were applied to each depletion column and incubated for 5 min at room temperature. The depleted serum was collected by centrifugation at 500 ×g for 10 sec. Protein concentrations of the depleted serum samples were measured with a Bradford assay (Bio-Rad Laboratories Inc., Hercules, CA).

#### 2.6.3. Tandem Mass Tag (TMT) Labeling

200 *μ*g depleted serum proteins from each group were precipitated in 20% trichloroacetic acid (TCA) at 4°C. Protein pellets were obtained by centrifugation (13,500 rpm), washed with ice-cold acetone, and resolubilized in 100 mM triethylammonium bicarbonate (TEAB) and 2,2,2-trifluoroethanol (TFE). Proteins were reduced with 10 mM tris(2-carboxyethyl)phosphine (TCEP) for 1 hour at 37°C and alkylated with 30 mM iodoacetamide (IAA) in the dark for 1 hour at room temperature. 2.5 *μ*g of mass spec grade trypsin/lysC (Promega, Madison, WI) was used to digest the peptides overnight at 37°C.

The digested peptides were quantified using Pierce™ Quantitative Colorimetric Peptide Assay (Thermo Scientific, Rockford, IL). 100 *μ*g of peptides from each specific sample was labeled with the Thermo Scientific™ TMTsixplex Isobaric Mass Tagging Kit (separately for female and male pools: day 0 with TMT^6^-126, day 2 with TMT^6^-127, day 7 ACV with TMT^6^-128, day 7 ACV + IVIG with TMT^6^-129, and day 7 PBS with TMT^6^-130) according to the manufacturer's protocol. The TMT^6^-131 label was used as a reference that contained 20 *μ*g of peptides from each of the five samples. All six labeled-peptide mixtures were combined into a single tube, mixed, and fractionated using the Thermo Scientific Pierce High pH Reversed-Phase Peptide Fractionation Kit. Nine fractionated samples were dried using the SpeedVac concentrator and resuspended in 1% formic acid before LC-MS/MS analysis. While this kit usually uses only eight fractions with step elutions of up to 50% acetonitrile, we added a ninth fraction eluting at 75% acetonitrile.

#### 2.6.4. LC-MS/MS Analysis

The samples were analyzed in an Orbitrap Fusion™ Tribrid™ mass spectrometer with the Thermo EASY-nLC™ ion source, 75 *μ*m × 2 cm Acclaim® PepMap100 C18 trapping column, and 75 *μ*m × 25 cm PepMap® RSLC C18 analytical column (Thermo). The column temperature was maintained at 45°C, and the peptides were eluted at a flow rate of 300 nL/min over a 130 min gradient, from 3–30% solvent B (100 min), 30–50% solvent B (3 min), 50–90% solvent B (2 min), and 90% solvent B (2 min). The solvent A was 0.1% formic acid in water and the solvent B was 0.1% formic acid in acetonitrile.

The full MS survey scan (m/z 400–1500) was acquired in the Orbitrap at a resolution of 120,000 and an automatic gain control (AGC) target of 2 × 10^5^. The maximum injection time for MS scans was 50 ms. Monoisotopic precursor ions were selected with charge states 2–7 and 70 s dynamic exclusion with a ± 10 ppm mass window. The MS^2^ scan (m/z 400–2000) was performed using the linear ion trap with the CID collision energy set to 35%. The ion trap scan rate was set to “rapid”, with an AGC target of 4 × 10^3^ and a maximum injection time of 150 ms. Ten fragment ions from each MS^2^ experiment were subsequently selected for an MS^3^ experiment. The MS^3^ scan (m/z 100–500) was performed to generate the TMT reporter ions in the linear ion trap using HCD at a collision energy setting of 55%, a rapid scan rate and an AGC target of 5 × 10^3^, and a maximum injection time of 250 ms.

#### 2.6.5. Data Analysis

All MS/MS spectra were searched using the Proteome Discoverer (version 2.1.0.81, Thermo Scientific) with searching engines Sequest-HT against the *Mus musculus* database (57,952 sequences). The searches were performed with the following parameters: 5 ppm tolerance for precursor ion masses and 0.6 Da tolerance for fragment ion masses. The static modification settings included carbamidomethyl of cysteine residues, and the dynamic modification included oxidation of methionine, TMT6plex modification of lysine *ε*-amino groups and peptide N-termini, and acetyl modification of peptide N-terminus. A target-decoy database search was used to obtain a false discovery rate (FDR) of 1%. The reporter ion integration tolerance was 0.5 Da. The total intensity of a reporter ion for a protein was calculated based on the sum of all detected reporter ions of associated peptides from that protein. The ratios between reporter ions and the reference reporter ion (TMT^6^-131) were used to estimate the abundance ratio of each protein.

Identified proteins were evaluated for upregulation and downregulation using a cutoff value of ≥1.5fold change. The gene ontology (GO) and KEGG pathway enrichment analysis were performed using the Database for Annotation, Visualization and Integrated Discovery (DAVID) v6.8 software (Nature Protocols, 2009). GO and KEGG pathway enrichment were analyzed with Fisher's exact test and corrected with the Benjamini-Hochberg procedure. Only pathways showing corrected *p* values ≤ 0.05 were considered significantly enriched.

### 2.7. Neurobehavioral Assessments

B6 mice infected bilaterally with HSV1 and treated with PBS, ACV, or ACV + IVIG at day 4 pi were monitored for a month before being transported to UC Davis for neurobehavior testing (aged 10 weeks). Male and female (*n* = 4/sex) healthy mice with no symptoms of HSE were included in each group. This study design was used to enable discovery of a large sex effect, but it was not suitable for detecting small sex effects.

#### 2.7.1. Elevated Plus Maze (EPM)

Mice were tested for anxiety using the EPM as previously described (Woods, Yasui). The EPM apparatus consisted of two open (30 × 5 × 0.25 cm^3^) and two closed (30 × 5 × 6 cm^3^) arms emanating from a central platform (5 × 5 cm^2^) to form a plus shape. The apparatus was made of white plastic and elevated to a height of 60 cm above the floor. Briefly, test mice were placed onto the central platform of the plus maze for 5 min of free exploration. Mouse behavior was recorded under dim red light by video camera (Sony Digital Handycam) and analyzed using the SMART tracking system (San Diego Instrument, San Diego, CA, USA). Distance traveled, number of entries, and duration of time spent in the open versus closed arms were recorded. Two uninfected (UN) mice were excluded from analysis because they inexplicably failed to move during testing.

#### 2.7.2. Morris Water Maze

Spatial learning and memory were tested in the Morris water maze using room cues as previously described [26, 27]. Mice were tested in cohorts of 4 at the same time of day (0900–1200) using a 90 cm diameter tank with 21°C water. Four 90 s trials per day were conducted for 4 days (males) or 5 days (females) because spatial learning is lower for females. After release from designated starting point, the mice had 90 sec to find a 6 × 6 cm^2^ platform 1 cm below the water surface made opaque with nontoxic paint powder. After reaching the platform or after 90 s elapsed, the mouse remained on the platform for 30 s and then was removed to a warming cage for 10–15 min intertrial interval. Training was preceded by a visible trial test (90 s) on the first day and followed by a probe trial (90 s) on the fifth day video tracking used the SMART system (V3.0, PanLabs, US distributor) which recorded latency and distance to reach the platform and time spent in each platform quadrant. Additionally, floating time was estimated by the tester. Testers were blind to the treatment groups of the mice.

#### 2.7.3. Statistical Analysis of Behavioral Data

A tiered approach was used to determine whether HSV infection caused behavioral impairment and whether this could be reversed by the treatments. First, statistical analysis (JMP, SAS Institute) compared the HSV-uninfected (UN) group and HSV-infected-untreated (PBS) group to determine which behavioral endpoints were sensitive to infection. These endpoints were also screened for sex effects. The different treatment groups were then individually compared to the PBS group on these endpoints to determine whether the HSV effect could be reversed. All pairwise (two-group) comparisons were conducted with one-way ANOVA or *F*-test using General Linear Modeling (JMP, SAS Institute).

## 3. Results

### 3.1. Mouse Model to Assess Behavior in Mice Surviving HSE

We previously reported that ACV protection of susceptible 129S6 (129) mice was reduced when treatment was initiated after day 2 pi because ACV failed to curtail the escalating CNS inflammatory responses [28]. In contrast, IVIG administered at 24 h pi protected all mice as did ACV [17]. However, there was a dramatic decline in protection when these drugs were given at progressively later times after infection, such that survival ranged from 15% for IVIG to 25% for ACV at day 4 pi (Fig. S1 in [17] and Fig. 4 in [28], resp.). Protection by ACV decreased over time primarily because it failed to restrain the escalating CNS inflammatory responses (Fig. S1 in [28]). In contrast, IVIG-mediated protection declined due to inflammatory innate immune cell infiltration into the CNS in the interim prior to IVIG administration (Fig. 4 in [17]). These data show that protection by ACV and IVIG against HSE depends critically on the timing of administration of the drug after infection.

B6 mice are highly resistant and do not normally develop HSE after unilateral ocular inoculation [17, 25, 29, 30]. However, bilateral inoculation of B6 mice, infected according to the scheme in Figure 1(a) with 1 × 10^5^ PFU per eye, resulted in CNS inflammation and HSE symptoms in all mice. Only ~65% of mice in the PBS group survived, compared to the 100% survival for mice treated with ACV alone or ACV + IVIG from day 4 pi (Figure 1(b)). Thus, delaying administration of ACV or ACV + IVIG to HSV-infected mice until day 4 pi allowed for CNS inflammation to develop, but not to an extent that resulted in excessive mortality from HSE. We also investigated uniocular inoculation, but the mice did not develop HSE or overt CNS inflammation, even when infected with the same total dose of virus (2 × 10^5^ PFU/mouse) as for the bilaterally inoculated mice. Therefore, we used bilaterally inoculated B6 mice for behavior testing, as it facilitated comparisons among PBS-, ACV-, and ACV + IVIG-treated mice that had experienced CNS inflammation.

**Figure 2:**
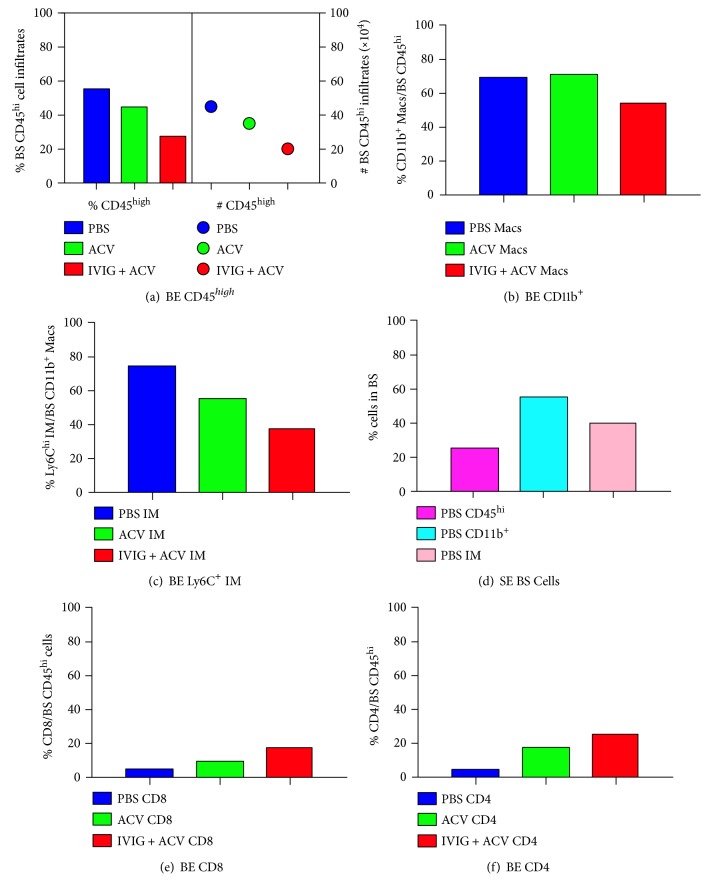
Inflammatory infiltrates in the BS of HSV1-infected B6 mice. Mononuclear cells isolated from BS of PBS- (blue), ACV- (green), or IVIG + ACV- (red) treated HSV1 bilaterally infected mice at day 7 pi were analyzed for (a) % CD45^hi^ infiltrates, left *y*-axis (bars) and absolute cell numbers, right *y*-axis (circles) and (b) CD45^high^ CD11b^+^ monocyte/macrophages. (c) CD45^high^ CD11b^+^ Ly6C^high^ IM. (d) BS cell subsets in HSV1 unilaterally infected mice. (e) CD8 and (f) CD4 T cells in the BS of bilaterally infected mice treated with PBS, ACV, or ACV + IVIG (*n* = 4-5 mice/group).

**Figure 3:**
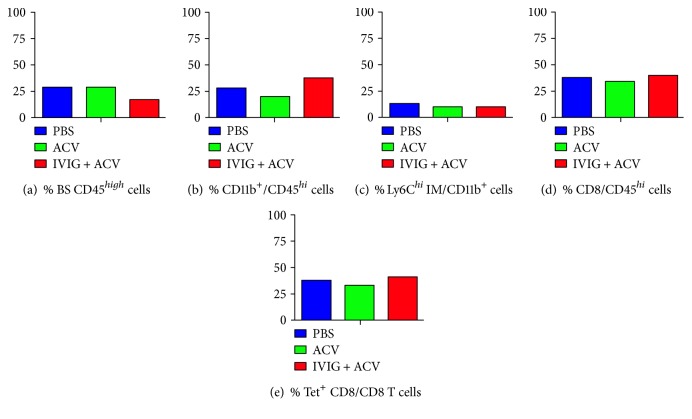
Inflammatory infiltrates in the BS of HSV1-infected B6 mice at day 21 pi. Mononuclear cells isolated at day 21 pi from BS of PBS- (blue), ACV- (green), or ACV + IVIG- (red) treated HSV1 bilaterally infected mice were analyzed for (a) CD45^high^ infiltrates, (b) CD45^high^ CD11b^+^ monocytes, (c) CD45^high^ CD11b^+^ Ly6C^high^ IM, (d) CD45^high^ CD8 T cells, and (e) CD45^high^ HSV-specific CD8 T cells (*n* = 4-5 mice/group).

We assessed inflammation in the brainstems of B6 mice bilaterally inoculated according to the scheme shown in Figure 1(a), and we included unilaterally inoculated PBS-treated mice for comparison. Brainstem (BS) infiltration of all immune cell subsets analyzed at day 7 pi was greater in bilaterally (both eyes: BE) inoculated mice compared to unilaterally (single eye: SE) inoculated mice, in terms of both % and absolute CD45^hi^ cell numbers (Figures 2(a), 2(b), 2(c), and 2(d)), consistent with greater susceptibility to HSE in the bilaterally inoculated mice. Compared to PBS-treated mice, combined ACV + IVIG treatment reduced BS inflammation to a greater extent than ACV alone and in particular the influx of Ly6C^high^ inflammatory monocytes at day 7 pi (Figure 2(c)), a pattern that persisted to day 21 pi (Figures 3(a), 3(b), and 3(c)). Interestingly, infiltration of CD4^+^ and CD8^+^ T cells was also increased by IVIG at day 7 pi (Figures 2(e) and 2(f)) and ~40% of infiltrating CD8^+^ T cells were HSV1 specific as determined by tetramer staining at day 21 pi (Figures 3(d) and 3(e); representative FACS plot shown in Supplementary Figure 1 available online at https://doi.org/10.1155/2017/5238402). Thus, the combination of ACV + IVIG imparts both antiviral and immunomodulatory activity.

### 3.2. Behavioral Assessment in Mice Surviving HSE

Exploratory behavioral phenotyping of the effects of HSV infection and response to ACV and IVIG therapies was conducted. Latently infected B6 male and female mice (*n* = 4/sex/group) treated with PBS, ACV, or ACV + IVIG according to the scheme outlined in Figure 1(a) were transferred to the UC Davis Mouse Biology Program phenotyping core 30–35 days pi and evaluated at day 45–50 pi for anxiety (EPM test) and spatial learning and memory (MWM test) using standardized protocols. Test parameters, schedule of testing, and endpoints measured are provided in Materials and Methods.

**Figure 4:**
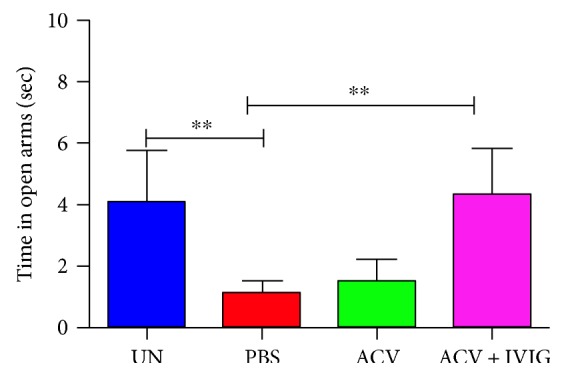
Elevated plus maze for anxiety in uninfected and HSV1-infected PBS-, ACV-, and ACV + IVIG-treated B6 mice. PBS versus uninfected: ^∗∗^*p* = 0.0019 and ACV + IVIG versus PBS: ^∗∗^*p* = 0.0014, *F*-test, *n* = 8 mice/group except for UN, *n* = 6.

Behavioral endpoints were first screened for HSV-induced deficits with the *F*-test to determine whether the test was sensitive to the HSV infection. The general level of anxiety, reflected by less time spent in the open arms of the EPM, was high for infected PBS- and ACV-treated B6 mice (Figure 4). Using the more rigorous one-way ANOVA (pairwise comparisons), the ACV + IVIG and PBS groups were also significantly different (*p* = 0.03). Importantly, combinatorial treatment with ACV + IVIG inhibited development of anxiety rendering the mice indistinguishable from uninfected mice.

**Figure 5:**
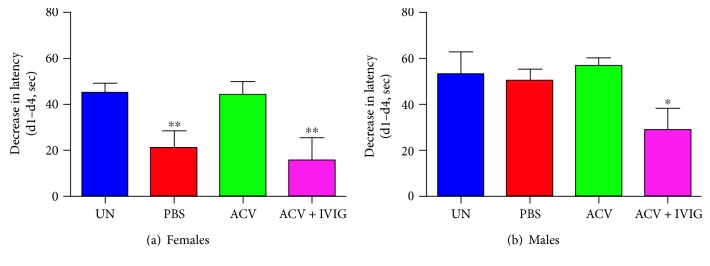
Morris water maze test to assess learning and memory in uninfected and HSV1-infected PBS-, ACV-, and ACV + IVIG-treated B6 mice. (*n* = 4 mice/sex/group). One-way analysis of variance (ANOVA): males ^∗^*p* = 0.0459, ACV + IVIG compared to uninfected (UN) group; females ^∗∗^*p* = 0.0075, ACV + IVIG and PBS compared to UN group.

HSV1 effects on spatial learning and memory (LM) were determined at the latent stage of infection (>day 45 pi) using the MWM, which is supported by extensive evidence as a valid measure of hippocampal-dependent spatial navigation and reference memory [31–33]. In contrast to anxiety (Figure 4), ACV treatment prevented learning deficits in female mice that were absent in male mice, revealing a sex-biased effect of HSV on behavior (Figure 5). Surprisingly, inclusion of IVIG antagonized the beneficial effects of ACV in female mice (Figure 5(a)), and the combination of ACV + IVIG also lead to poorer MWM learning in male (Figure 5(b)).

After corneal inoculation of one eye, HSV1 spreads to the trigeminal ganglia (Tg) and BS but it does not spread into the brains of B6 mice [34]. However, since we used bilateral inoculation, it was important to determine whether HSV1 spread into the brain. Infectious virus was absent from the cerebrum in both male and female mice at day 4 pi and either absent or present at very low levels (1–3 PFU) in both sexes at day 6 pi and cleared by day 8 pi as determined by plaque assay [35].

### 3.3. Changes in Serum Cytokines/Chemokines and the Proteome

**Figure 6:**
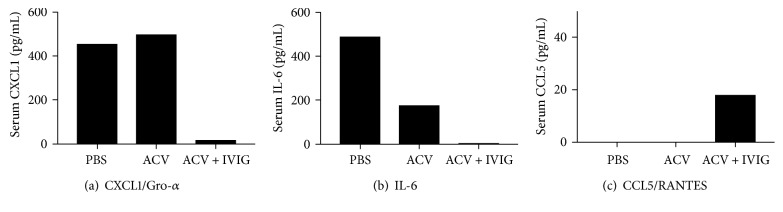
Cytokine and chemokine expression in infected B6 mice treated with PBS, ACV, or ACV + IVIG. (a) CXCL1/Gro-*α*, (b) IL-6, and (c) CCL5/RANTES were determined in sera obtained day 7 pi from the 3 groups of mice using a multiplex ELISA (*n* = 4/group, pooled serum samples).

A Luminex bead-based assay of serum samples collected from 2 M and 2 F mice on day 7 pi revealed differential expression of several cytokines and chemokines in the PBS-, ACV-, and ACV + IVIG-treated mouse groups, some of which are involved in egress of neutrophils and monocytes from the bone marrow and trafficking to site inflammation as observed in this study (Figures 6(a), 6(b), and 6(c) and Supplementary Figure 2). These include CXCL1 (Gro-*α*), CCL2, CCL7 (MCP-1, MCP-3), and CCL5 (RANTES), which have also been implicated in various behavioral impairments including anxiety, depression, and LM [36–38]. In a recent study of LM in rats of different ages, serum CXCL1/Gro-*α* and CCL5/RANTES levels were significantly elevated in aged, memory-impaired rats compared to “elite agers”; hence, they were considered viable candidate biomarkers for age-related memory loss [39]. Interestingly, CXCL1/Gro-*α* was elevated in the PBS- and ACV-treated mice but was suppressed in the ACV + IVIG-treated mice (Figure 6(a)). This was consistent with increased IM and neutrophil infiltration in the PBS- and ACV-treated groups. However, CCL5/RANTES levels were elevated in the ACV + IVIG-treated mice compared ACV- and PBS-treated mice (Figure 6(c)), which fits with the increased T cell infiltration in the BS of ACV + IVIG-treated mice (Figures 2(e) and 2(f)). Elevated levels of CXCL1/Gro-*α*, G-CSF, IL-6, TNF*α*, and CCL2/MCP-1 (Supplementary Figure 2) suggest a myeloid inflammatory phenotype in the PBS-treated mice concordant with increased CD11b^+^ and Ly6C^high^ IMs in the BS of PBS mice (Figures 2(b) and 2(c); only representative, functionally relevant cytokines are shown in Figure 6**)**.

About 300 and 500 proteins matching the *Mus musculus* database were detected in male and female serum samples, respectively. After normalizing the data to uninfected mice (day 0), about 16% of the total serum proteins in male mice and 7% of the total proteins in female mice were differentially expressed in each group. Identified proteins were evaluated for upregulation and downregulation using a cutoff value of ≥1.5fold change. The gene ontology (GO) and KEGG pathway enrichment analysis were performed using the Database for Annotation, Visualization and Integrated Discovery (DAVID) v6.8 software (Nature protocols, 2009). GO and KEGG pathway enrichment were analyzed with Fisher's exact test and corrected with the Benjamini-Hochberg procedure. Only pathways showing *p* values ≤ 0.05 were considered significantly enriched. A total of 21 male and only one female GO biological process categories were enriched relative to serum proteins in uninfected mice (day 0, Supplementary Table 1).

**Figure 7:**
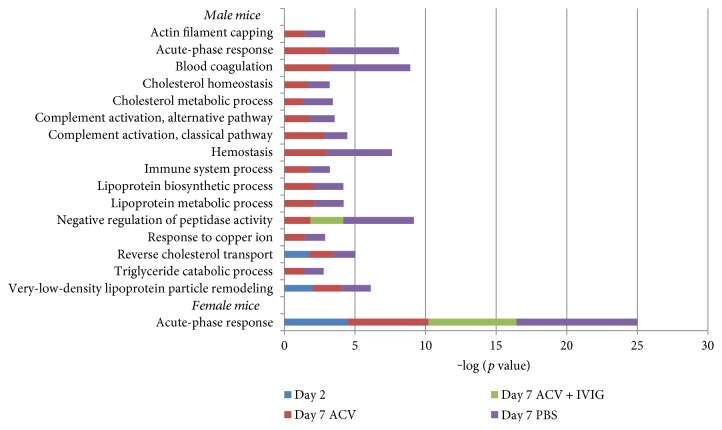
GO biological process analysis on the differentially expressed proteins in male and female mice according to each experimental condition. 15 GO categories with an enrichment in at least two conditions were selected.

**Figure 8:**
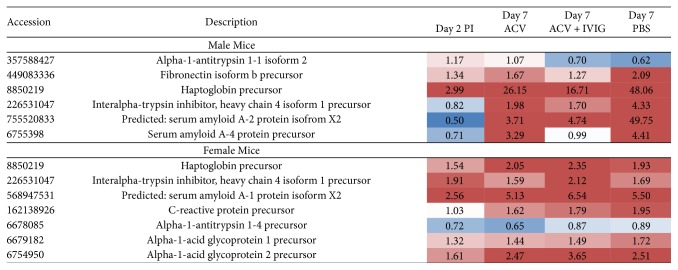
Heatmap representation of upregulated and downregulated proteins involved in the GO acute phase response (GO:0006953). Upregulation (red); downregulation (blue). The values represent the protein's abundance ratios normalized to those of uninfected mice.

**Figure 9:**
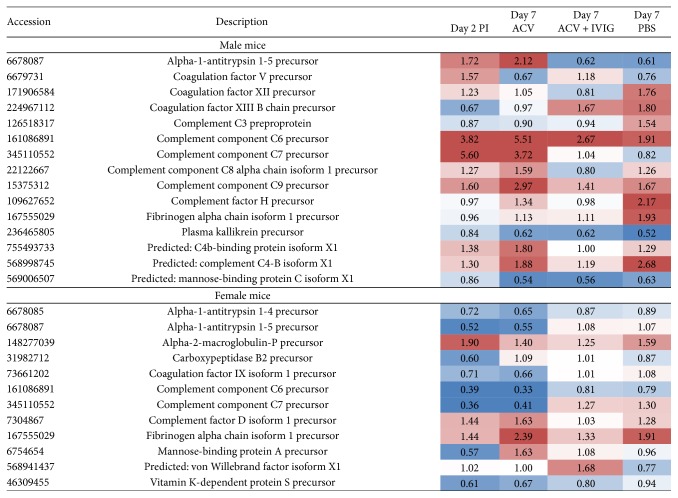
Heatmap representation of upregulated and downregulated proteins involved in KEGG complement and coagulation cascades. Upregulation (red); downregulation (blue). The values represent the protein's abundance ratios normalized to those of uninfected mice.

Several GO categories, such as acute-phase response, immune system process, cholesterol metabolism, and complement activation were enriched in male whereas only one of the acute-phase response GO category was enriched in female mice (Figure 7). However, the male mice were predominantly enriched in the inflammation category “acute-phase response,” manifested by upregulated serum amyloid proteins and haptoglobin (Figure 8). Moreover, KEGG pathway analysis showed consistent enrichment of coagulation and complement cascades in both male and female mice (Figure 9). Interestingly, complement components C6, C7, C8, and C9, responsible for membrane attack complex formation, were significantly upregulated in male mice on day 2 and after ACV treatment, but downregulated in the ACV + IVIG and the no-treatment groups (PBS). In contrast, those same complement proteins were downregulated in female mice on day 2 and after ACV treatment compared to the ACV + IVIG treatment group. Furthermore, protein *α*-1-antitrypsin 1-5 precursor (A1AT, GI:6678087), an important proinflammatory protein, was significantly upregulated in male mice on day 2 and after ACV treatment compared to after ACV + IVIG or no treatment (PBS). However, A1AT was downregulated in female mice on day 2 and after ACV treatment (Figure 9).

Acute and prolonged activation of complement proteins (C3a, C3b, C5, and C5a) is seen in CSF of patients with HSE but not healthy controls [40]. Notably, only infected male PBS-treated mice died (Figure 1), consistent with our report of a sex difference in genetic resistance to HSE [41]. Complement C3a was implicated in memory loss in a model of WNV CNS infection [42] and intriguingly has also been shown to stimulate neurogenesis in adult mice and promotes faster functional recovery after ischaemic brain injury [43, 44], indicative of dual deleterious and protective roles.

Importantly, ACV and ACV + IVIG had opposite effects on expression of some of the complement proteins in male and female mice at day 7 pi relative to PBS, for example, complement C7 precursor (Figure 9). Serum amyloid A-4 protein precursor was absent in female mice but highly upregulated in male ACV- and PBS-treated mice compared to ACV + IVIG-treated mice (Figure 8).

Acute serum amyloid (A-SAA) proteins are danger signals that serve as endogenous ligands for TLR2 and TLR4. HSV1 infection induced rapid expression of A-SAA resulting in potentiated inflammatory responses [45]; thus, A-SAA and complement proteins are candidate biomarkers for HSE. Although we cannot discern specific proteins that might be implicated in the LM deficits in F but not M mice from this analysis, the data tend to discount heightened expression of inflammatory response proteins (>M than F) associated with HSE as being involved. These observed gender-specific changes of the aforementioned serum proteins go hand in hand with the observed differences in behavior of male and female mice after HSV infections and with treatments.

## 4. Discussion

A majority of patients (>60%) surviving HSE suffer neurological disturbances that can include loss of smell (anosmia) and speech (aphasia), anxiety, and learning and memory impairments that can be severe despite antiviral treatment [3, 46]. In contrast, ~70% of neonates surviving HSV1 infection suffer permanent neurological impairment, exhibiting diverse symptoms including motor abnormalities such as hemiparesis and spastic quadriplegia, delayed speech, visual impairment or blindness, recurring seizures, and microencephally [47, 48]. The pathophysiological mechanisms responsible for neurologic dysfunction following HSV1 CNS infection are largely unknown. However, the finding that poor neurological outcomes are highly correlated with delayed ACV treatment has been interpreted as evidence that viral-replication-triggered events are casually involved [2].

We exploited delayed administration of antiviral and/or anti-inflammatory drugs to develop a novel mouse model to facilitate studies to elucidate the mechanisms underlying neurological dysfunction resulting from HSV1 CNS infection. Following bilateral ocular infection, all resistant B6 mice developed HSE symptoms and brainstem inflammation that remarkably were absent in mice unilaterally inoculated with the same dose of virus. Although, currently, we cannot explain this difference in pathogenesis, bilateral inoculation of B6 mice nonetheless allowed us to address the role of CNS inflammation in HSV1-induced neurological deficits.

Mice treated combinatorially with ACV + IVIG were free of anxiety compared to ACV-treated and PBS-treated infected mice that exhibited high levels of anxiety as determined using the elevated maze plus test. The open field test to assess anxiety revealed a similar trend for females but not males, but given the low number of mice per sex (*n* = 4), statistical significance was not achieved (not shown). IVIG-mediated protection against anxiety was correlated with reduced brainstem inflammation and in particular influx of Ly6C^high^ inflammatory monocytes during acute infection (day 7 pi), which we previously reported were casually involved in HSE [17]. This correlation is consistent with the recent report that peripheral suppression of Ly6C^high^ inflammatory monocyte infiltration into the CNS mitigated neuroinflammation in several brain regions and the development of anxiety in models of inflammation and psychosocial stress induced behavioral abnormalities [49]. Indeed, evidence from other rodent models, including repeated social defeat models, corroborates monocyte trafficking into the brain as being casually involved in establishment of anxiety-like behaviors in response to chronic stress [50]. The recent report that genetic- or antibiotic-mediated depletion of Ly6C^high^ monocytes decreased neurogenesis and memory retention suggests that the Ly6C^high^ monocyte population is fundamental for brain homeostasis and plasticity [51]. Thus, Ly6C^high^ monocytes can exert either detrimental or beneficial effects depending on the host inflammatory status, with inflammatory Ly6C^high^ monocytes exerting destructive effects that though beneficial for pathogen clearance can cause host tissue destruction if not tightly controlled [17, 51–55].

The observed sex-biased effects of HSV on learning and memory and the beneficial and detrimental effects, respectively, of ACV and IVIG on behavior are difficult to ascribe to direct effects of HSV infection of brain cells. The hippocampus is the primary brain region involved with learning and memory [56, 57]. HSV1-inflicted damage to hippocampal neurons is discounted as the cause of learning and memory deficits because infectious HSV was either absent or present only at very low but equivalent levels in the brains of both male and female mice during acute infection. Thus, these data raise several important questions including (i) why are learning and memory impairments confined to female mice? (ii) how does ACV prevent cognitive decline, given that it is a guanosine nucleoside analogue that purportedly preferentially inhibits viral DNA replication? [58, 59] and (iii) how does IVIG antagonize the effects of ACV, as we have shown that it is excluded from the brain and rather acts peripherally to exert anti-inflammatory activities that protect against HSE [17]. In a recent phase III trial with 390 subjects, the Gammaglobulin Alzheimer's Partnership (GAP) study, IVIG treatment failed to meet the primary endpoints of slowing cognitive and functional decline after 18 months of treatment, but no adverse effects on behavior were reported [60]. Ganciclovir (GCV), a nucleoside prodrug analogue of ACV, was recently reported to be a potent inhibitor of microglial proliferation that effectively inhibited experimental autoimmune encephalomyelitis (EAE) by reducing the infiltration of CD11b^+^ CD45^high^ Iba^+^ macrophages and T cells into the CNS [61]. In our study, ACV treatment from day 4 pi reduced infiltration of monocytes especially the Ly6C^high^ subset into the brainstem, but whether inhibition of CNS microglia proliferation occurs warrants further study.

An earlier study implicated that HSV1 induced inflammation and lesions in the cortex and brainstem with the development of severe spatial memory deficits in female mice [13]. No other behavioral traits were examined in that study that also excluded male mice, which is unfortunate as it is only rodent study that directly measured HSV1 effects on learning and memory. Perturbation of neural stem cell (NSC) neurogenesis in the dentate gyrus of the hippocampus is increasingly being associated with cognitive deficits, including spatial memory and particularly long-term memory retention in the Morris water maze test [62, 63]. Recent studies have shown that some peripheral illnesses and metabolic diseases as well as aging result in chronic inflammation that is associated with behavioral impairments, including anxiety, depression, and learning and memory deficits that have been linked to disrupted neurogenesis [56]. Of interest in this context are recent reports that HSV1 infection, which causes chronic inflammation in the brain [35, 64, 65], can result in impaired neurogenesis [66, 67]. An intriguing observation is that activated HSV1-specific CD8 T cells can suppress NSC proliferation in *in vitro* cocultures via interaction of secreted IFN-*γ* with IFN-*γ*R on NSCs [68]. We and others documented long-term persistence of activated HSV1-specific T cells associated with production of IFN-*γ* in sensory ganglia and the brains of mice and humans, which raises the possibility that impaired neurogenesis during latency might be involved in HSV1-induced cognitive decline [35, 64, 69]. Hippocampal learning and memory have recently been shown to depend on Wnt signaling, and the Wnt signaling pathway is known to be influenced by stress and is responsive to sex hormones [57, 70]; hence, Wnt signaling in NSCs may be involved in sex-biased learning and memory deficits in HSV1-infected mice. As ACV appears to target activated proliferating cells in specific brain regions [61], it will be important to determine in future studies if its protective effects against learning and memory deficits in B6 female mice involve effects on microglia/infiltrating CD45^high^ cells.

Overall, the most important finding from this exploratory study is that ACV can protect against cognitive impairment even when given 4 days pi though its mechanism of action is uncertain and likely novel. Interestingly in a recent clinical trial, prolonged oral dosing of ACV (6 months) following standard 14-day intravenous ACV was reported to dramatically improve neurological outcomes for neonates with HSV CNS involvement [47]. In contrast, in a more recent trial based on the success with neonates, prolonged Valacyclovir treatment showed no benefit in adult HSE patients and even worsened cognitive outcomes for some patients [14]. Anxiety, in contrast to learning and memory, was associated with CNS inflammation and thus mitigated by IVIG's anti-inflammatory effects. The negative impact of combinatorial treatment with ACV + IVIG on learning and memory is interesting and important given the widespread clinical use of IVIG in combination with ACV/GCV patients undergoing hematopoietic stem cell transplantation for hematologic malignancies [71]. Although, the results of this study raise more questions than answers, our experimental mouse HSE model is ideal for investigating how ACV and IVIG affect cognition when given alone or in combination. Moreover, the antagonistic effects of ACV and IVIG on learning and memory provide an opportunity to identify biomolecules that positively or negatively impact behavior.

## Supplementary Material

Supplementary Figure 1. Representative FACS plots of brainstems isolated from the 3 groups of mice at day 21 pi showing mononuclear cells expressing (A) CD45high infiltrates, (B) CD11b+ monocytes, (C) Ly6C expressing IM, (D) HSV gB498 specific CD8 T cells showing reactivity for gB498-505 tetramer; (*n*=4 − 5 mice/group). Supplementary Figure 2. Serum cytokines and chemokines in the 3 groups of mice at day 7 pi. Sera from 4 mice / group were analyzed for G-CSF, CCL2, TNF and IL-23 by luminex multiplex assay. Supplementary Table 1: GO biological process categories enriched in serum proteins of HSV infected male and female mice.
