# Supplementary material for: Effects of Acyclovir and IVIG on Behavioral Outcomes after HSV1 CNS Infection
Source: Behav Neurol. 2017 Nov 19;2017:5238402. doi: 10.1155/2017/5238402 (PMC5735307; doi:10.1155/2017/5238402)
Supplement: Supplementary file 1 — Supplementary Figure 1. Representative FACS plots of brainstems isolated from the 3 groups of mice at day 21 pi showing mononuclear cells expressing (A) CD45high infiltrates, (B) CD11b+ monocytes, (C) Ly6C expressing IM, (D) HSV gB498 specific CD8 T cells showing reactivity for gB498-505 tetramer; (n=4 − 5 mice/group). Supplementary Figure 2. Serum cytokines and chemokines in the 3 groups of mice at day 7 pi. Sera from 4 mice / group were analyzed for G-CSF, CCL2, TNF and IL-23 by luminex multiplex assay. Supplementary Table 1: GO biological process categories enriched in serum proteins of HSV infected male and female mice. [file 5238402.f1.pdf]

Supplementary Figure 1.

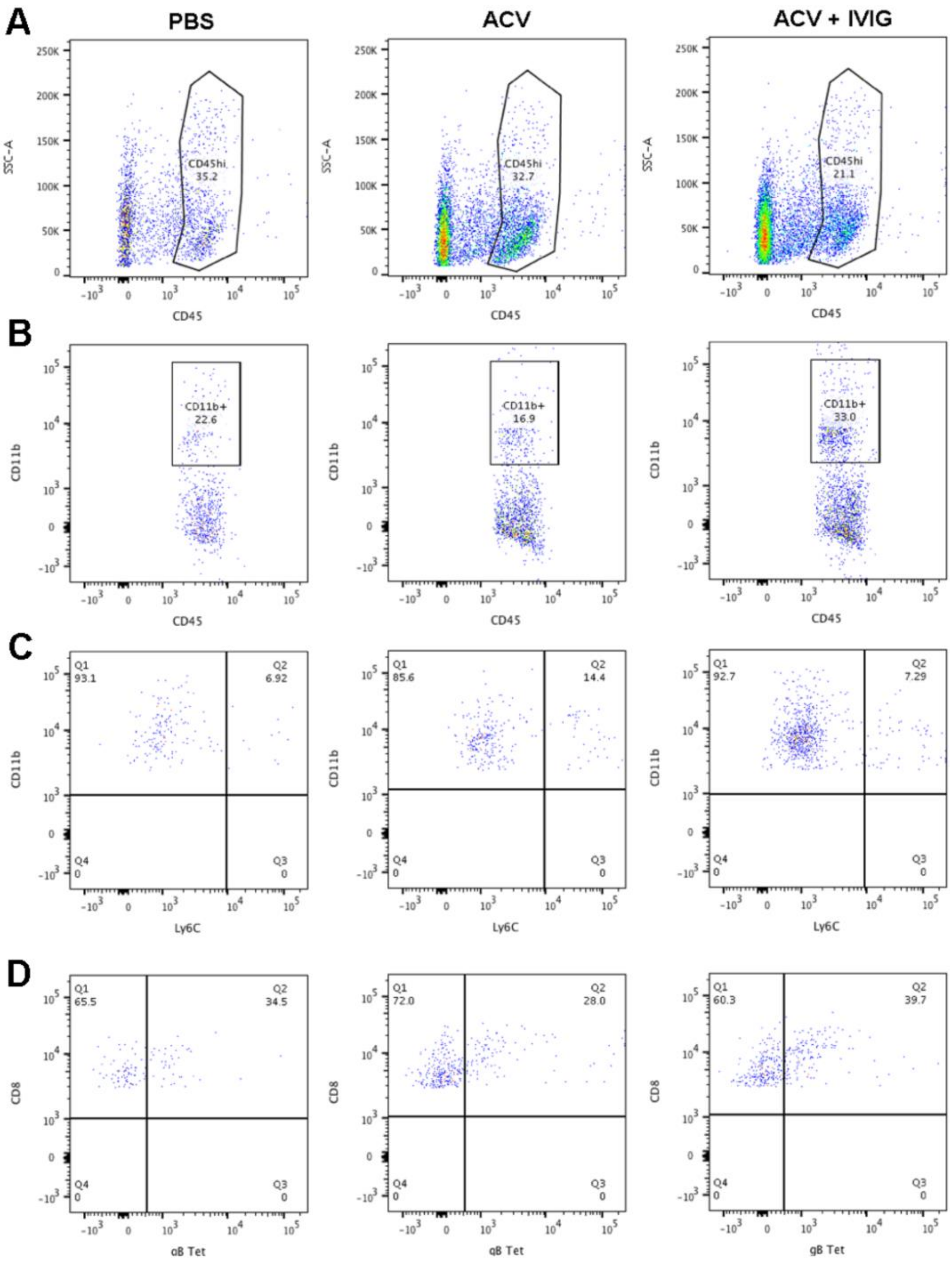

Suppl Fig. 1. Representative FACS plots of brainstems isolated from the 3 groups of mice at day 21 pi showing mononuclear cells expressing (A) $CD45^{high}$ infiltrates, (B) CD11b+ monocytes, (C) Ly6C expressing IM, (D) HSV gB_{498} specific CD8 T cells showing reactivity for $gB_{498\text{-}505}$ tetramer; (n=4-5 mice/group).

Supplementary Figure 2.

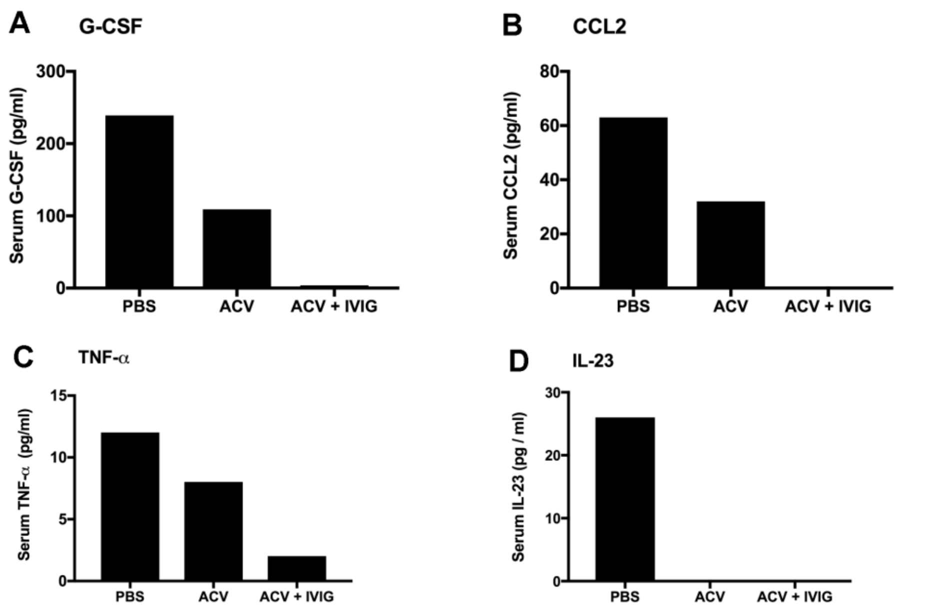

Suppl. Fig. 2. Serum cytokines and chemokines in the 3 groups of mice at day 7 pi. Sera from 4 mice / group were analyzed for G-CSF, CCL2, TNF and IL-23 by luminex multiplex assay.

GO: Biological Process					
**Term**	**Count**	**%**	**P-Value**	**Benjamini**	**–log(p-value*)**
**Male: Day 2 PI**					
very-low-density lipoprotein particle remodeling	3	10.7	3.00E-05	9.00E-03	2.05
reverse cholesterol transport	3	10.7	1.10E-04	1.70E-02	1.77
**Male: Day 7 ACV**					
blood coagulation	6	11.1	2.50E-06	6.10E-04	3.21
acute-phase response	5	9.3	2.00E-06	9.50E-04	3.02
hemostasis	5	9.3	6.00E-06	9.70E-04	3.01
complement activation, classical pathway	5	9.3	1.20E-05	1.50E-03	2.82
lipoprotein metabolic process	4	7.4	7.70E-05	7.40E-03	2.13
lipoprotein biosynthetic process	3	5.6	9.90E-05	7.90E-03	2.10
very-low-density lipoprotein particle remodeling	3	5.6	1.40E-04	9.40E-03	2.03
negative regulation of peptidase activity	5	9.3	2.40E-04	1.40E-02	1.85
complement activation, alternative pathway	3	5.6	2.90E-04	1.60E-02	1.80
immune system process	7	13	4.50E-04	2.10E-02	1.68
reverse cholesterol transport	3	5.6	5.10E-04	2.20E-02	1.66
phosphatidylcholine metabolic process	3	5.6	5.10E-04	2.20E-02	1.66
cholesterol homeostasis	4	7.4	5.90E-04	2.30E-02	1.64
actin filament capping	3	5.6	9.90E-04	3.60E-02	1.44
response to copper ion	3	5.6	9.90E-04	3.60E-02	1.44
cholesterol transport	3	5.6	1.20E-03	4.10E-02	1.39
triglyceride catabolic process	3	5.6	1.20E-03	4.10E-02	1.39
cholesterol metabolic process	4	7.4	1.60E-03	5.00E-02	1.30
**Male: Day 7 ACV+IVIG**					
negative regulation of peptidase activity	5	15.2	2.20E-05	4.80E-03	2.32
**Male: Day 7 PBS**					
blood coagulation	8	13.8	4.10E-09	2.00E-06	5.70
acute-phase response	6	10.3	4.90E-08	8.00E-06	5.10
negative regulation of peptidase activity	8	13.8	4.20E-08	1.00E-05	5.00
hemostasis	6	10.3	2.00E-07	2.50E-05	4.60
response to cytokine	6	10.3	3.50E-06	3.40E-04	3.47
cholesterol metabolic process	5	8.6	1.20E-04	7.50E-03	2.12
lipoprotein biosynthetic process	3	5.2	1.20E-04	8.40E-03	2.08
lipoprotein metabolic process	4	6.9	1.00E-04	8.50E-03	2.07
very-low-density lipoprotein particle remodeling	3	5.2	1.70E-04	9.10E-03	2.04
complement activation, alternative pathway	3	5.2	3.60E-04	1.70E-02	1.77
complement activation, classical pathway	4	6.9	5.30E-04	2.30E-02	1.64
reverse cholesterol transport	3	5.2	6.20E-04	2.50E-02	1.60
cholesterol homeostasis	4	6.9	7.90E-04	2.70E-02	1.57
fibrinolysis	3	5.2	8.30E-04	2.70E-02	1.57
immune system process	7	12.1	7.80E-04	2.90E-02	1.54
response to peptide hormone	4	6.9	1.20E-03	3.40E-02	1.47
actin filament capping	3	5.2	1.20E-03	3.60E-02	1.44
response to copper ion	3	5.2	1.20E-03	3.60E-02	1.44
triglyceride catabolic process	3	5.2	1.50E-03	4.00E-02	1.40
**Female: Day 2 PI**					
acute-phase response	4	33.3	5.50E-07	3.30E-05	4.48
**Female: Day 7 ACV**					
acute-phase response	5	27.8	1.20E-08	1.80E-06	5.74
**Female: Day 7 ACV+IVIG**					
acute-phase response	5	35.7	5.80E-09	6.30E-07	6.20
**Female: Day 7 PBS**					
acute-phase response	6	37.5	2.60E-11	2.70E-09	8.57

Supplementary Table 1: GO biological process categories enriched in serum proteins of HSV infected male and female mice.
